# ﻿An updated list of the Mexican herpetofauna: with a summary of historical and contemporary studies

**DOI:** 10.3897/zookeys.1166.86986

**Published:** 2023-06-12

**Authors:** Aurelio Ramírez-Bautista, Lizzeth A. Torres-Hernández, Raciel Cruz-Elizalde, Christian Berriozabal-Islas, Uriel Hernández-Salinas, Larry David Wilson, Jerry D. Johnson, Louis W. Porras, Carlos Jesús Balderas-Valdivia, Adriana J. X. González-Hernández, Vicente Mata-Silva

**Affiliations:** 1 Laboratorio de Ecología de Poblaciones, Centro de Investigaciones Biológicas, Instituto de Ciencias Básicas e Ingeniería, Universidad Autónoma del Estado de Hidalgo, Km 4.5 Carretera Pachuca-Tulancingo, 42184 Mineral de La Reforma, Hidalgo, Mexico Universidad Autónoma del Estado de Hidalgo Mineral de la Reforma Mexico; 2 Laboratorio de Ecología y Diversidad Faunística, Facultad de Ciencias Naturales, Universidad Autónoma de Querétaro, Avenida de las Ciencias S/N, Santa Fe Juriquilla, C. P. 76230, Querétaro, Querétaro, Mexico Universidad Autónoma de Querétaro Querétaro Mexico; 3 Programa Educativo de Ingeniería en Biotecnología, Universidad Politécnica de Quintana Roo, Av. Arco Bicentenario, M 11, Lote 1119-33, Sm 255, 77500 Cancún, Quintana Roo, Mexico Universidad Politécnica de Quintana Roo Cancún Mexico; 4 Instituto Politécnico Nacional, CIIDIR Unidad Durango, Sigma 119, Fraccionamiento 20 de Noviembre II, Durango 34220, Mexico Instituto Politécnico Nacional, CIIDIR Unidad Durango Durango Mexico; 5 Centro Zamorano de Biodiversidad, Escuela Agrícola Panamericana Zamorano, Departamento de Francisco Morazán, Tegucigalpa, Honduras Centro Zamorano de Biodiversidad, Escuela Agrícola Panamericana Zamorano Tegucigalpa Honduras; 6 1350 Pelican Court, Homestead, Florida 33035-1031, USA Unaffiliated Homestead United States of America; 7 Department of Biological Sciences, The University of Texas at El Paso, El Paso, Texas 79968-0500, USA The University of Texas at El Paso El Paso United States of America; 8 7705 Wyatt Earp Avenue, Eagle Mountain, Utah, 84005, USA Unaffiliated Eagle Mountain United States of America; 9 Dirección General de Divulgación de la Ciencia, Zona Cultural de Ciudad Universitaria, Universidad Nacional Autónoma de México, Mexico City, Mexico Universidad Nacional Autónoma de México Mexico City Mexico; 10 Colección Nacional de Anfibios y Reptiles, Instituto de Biología, Universidad Nacional Autónoma de México, Ciudad Universitaria, Coyoacán, CP04510, Mexico Universidad Nacional Autónoma de México Coyoacán Mexico

**Keywords:** Amphibians, Mexico, reptiles, species diversity

## Abstract

The growth in our knowledge of the diversity of the herpetofauna of Mexico has occurred over the period of approximately 445 years from the work of Francisco Hernández to that of a broad multinational array of present-day herpetologists. The work of this huge group of people has established Mexico as one of the most significant centers of herpetofaunal biodiversity in the world. This status is the result of a complex orography, in addition to diverse habitats and environments and the biogeographic history of Mexico. The current herpetofauna consists of 1,421 native and introduced species, allocated to 220 genera, and 61 families. This figure is comprised of 1,405 native species and 16 non-native species (as of April 2023). The non-native species include two anurans, 13 squamates, and one turtle. The level of endemism is very high, presently lying at 63%, with this level expected to increase with time. Species richness varies among the 32 federal entities in the country, from a low of 50 in Tlaxcala to a high of 492 in Oaxaca. Amphibian species richness by state-level can be envisioned as comprising three levels of low, medium, and high, with the lowest levels occurring in the Peninsula of Baja California, a group of seven states in north-central and central Mexico, and a group of three states in the Yucatan Peninsula, with the highest levels occupying the southern states of Guerrero, Puebla, Veracruz, Oaxaca, and Chiapas, and the medium level in the remaining states of the country. Reptile species richness also can be allocated to three categories, with the lowest level occupying Baja California Sur, a group of central states, and the states of the Yucatan Peninsula, and the highest level found in a cluster of the states of Veracruz, Guerrero, Oaxaca, and Chiapas. Knowledge of the Mexican herpetofauna will continue to grow with additional studies on systematics, conservation, and the construction of checklists at various levels.

## ﻿Introduction

Historically, our knowledge of the diversity of different biological groups in Mexico has increased through the accumulation of local, regional, state, and/or country level studies (mammals: Sánchez-Cordero et al. 2014, birds: Navarro-Sigüenza et al. 2014; amphibians: Wilson et al. 2010, 2013a; Parra-Olea et al. 2014; and reptiles Wilson et al. 2010, 2013b; Flores-Villela and García-Vázquez 2014; Ramírez-Bautista et al. 2014). In studying history of the Mexican herpetofauna, the amount of species richness in each area has undergone two major stages: one in which it advanced rapidly, but slowed after the publication of Smith and Taylor (1966), and the other involving the integration of local studies that provided information for cataloging species at the regional level. At the regional or state levels, however, the lists initially were anecdotal, which resulted in a broad gap between the actual records and the first official herpetofaunal catalogs (as reprinted in Smith and Taylor 1966).

The contributions of Smith and Smith (1976, 1977, 1993) laid the foundation for a series of studies aimed at determining the species richness of amphibians and reptiles. Importantly, the growing number of amphibian and reptile species and subspecies noted by these authors was highly significant. In recent years, most of the species’ richness has been assessed at the species level, based on the dubious legitimacy of subspecies as a formal taxonomic hierarchical category (Flores-Villela et al. 2004; Wilson et al. 2010; Johnson et al. 2015). Nonetheless, the number of species keeps increasing, based on the rate of taxonomic changes, the description of new species, rediscoveries, and the resurrection of species (Lara-Tufiño et al. 2016; Nicholson et al. 2018; Nieto-Montes de Oca et al. 2018; Mata-Silva et al. 2019). An increasing amount of species richness has also resulted from the continuing amount of work being conducted in several parts of the country. In addition to the information provided by Smith and Taylor (1966), Álvarez del Toro (1982) reported on the conservation of biodiversity at both the regional and state levels (Flores-Villela et al. 2004). Prior to the publication of Smith and Taylor (1966), there were various studies on the description of species and subspecies, but there were few studies aimed at listing species by region, and their contribution was the first to provide information on species richness (a list of species), keys for determining species, synonymies, and information on geographic distribution. During this period, an interest in these groups was minimal based on the low number of herpetologists in Mexico, and because institutions that promoted field research had limited (or non-existing) funds to print catalogs at regional and state levels (Pérez-Higareda and Smith 1991).

Finally, between 1990 and 2000, a strong interest in biodiversity led to an assessment of the herpetofauna of Mexico at both the regional and state levels (Flores-Villela and Canseco-Márquez 2004; Wilson et al. 2013a, b). Subsequently, information from these projects, along with long-term studies and continued sampling, provided a more accurate number of the species involved. These studies were followed by larger ones, which included a greater number of states (Vázquez-Díaz and Quintero Díaz 1997, 2005; Lemos-Espinal and Smith 2009; Dixon and Lemos-Espinal 2010; Lemos-Espinal and Dixon 2013; Ramírez-Bautista et al. 2014; among others), as well as those from the entire country (Flores-Villela and Canseco-Márquez 2004; Wilson and Johnson 2010; Wilson et al. 2013a, b; Johnson et al. 2017). Presently, these studies form the basis of our knowledge of the biodiversity of amphibians and reptiles in Mexico (Wilson and Johnson 2010; Wilson et al. 2013a, b; Johnson et al. 2017; Balderas-Valdivia and González-Hernández 2021; Balderas-Valdivia et al. 2022; Smith and Lemos-Espinal 2022).

Flores-Villela (1993a) reported the number of species in Mexico based primarily on the published literature and provided an analysis of the Mexican herpetofauna based on Smith and Taylor (1966) and Smith and Smith (1976, 1977, 1993). Additionally, Flores-Villela (1993b) noted the number of amphibian and reptile species as 291 and 706, respectively; these numbers eventually changed to 361 and 804 species, respectively (1,165 species; Flores-Villela and Canseco-Márquez 2004). In time, Liner (2007) provided an updated list that included both species and subspecies and indicated 393 amphibians and 1,234 reptiles. The remarkable species richness reported in these studies was related to several factors, such as the complex physiography of Mexico, the representation of both Nearctic and Neotropical regions in the country, as well as its diverse biogeographic regions and vegetational formations (Morrone 2005). Subsequent knowledge of the herpetofauna was analyzed in the context of its diversity, in which its components are the richness and abundance of species (Ochoa-Ochoa et al. 2014), and how this might vary according to environmental parameters (Cruz-Elizalde et al. 2016). Similar numbers of amphibians (376) and reptiles (864) were reported by Parra-Olea et al. (2014) and Flores-Villela and García-Vázquez (2014), respectively; however, more recent studies showed an increase to 1,395 native species (394 amphibians, and 898 reptiles; Johnson et al. 2017; Balderas-Valdivia et al. 2022). These studies did not consider any introduced species and should be updated with recent taxonomic changes and new species and records (i.e., Hidalgo-García et al. 2023). Additionally, increases in the richness and diversity of the herpetofauna is constantly monitored (i. e. Herpetología Mexicana 2023; Mesoamerican Herpetology Taxonomic List 2023) but they do not provide a historical analysis of the richness or the proportion of state herpetofaunal biodiversity.

The increase of species richness reflects the systematic studies conducted by both Mexican and foreign herpetologists during the last three decades, in addition to the impact of the previously mentioned historical studies. Therefore, with the descriptions of new species, the knowledge of the biodiversity of Mexico is enhanced, as well as attention to its conservation needs (Campbell et al. 2018; Mata-Silva et al. 2019). This statement is supported by recent studies in which several species were described, e.g., the salamanders *Chiropterotritonaureus* or *C.nubilus* (García-Castillo et al. 2018), the lizards *Gerrhonotusmccoyi* (García-Vázquez et al. 2018a), and *Xenosaurusfractus* (Nieto-Montes de Oca et al. 2018), snakes such as *Rhadinaeanuchalis* (García-Vázquez et al. 2018b) and *Geophiscansecoi* (Grünwald et al. 2021), among others, and even the descriptions of new snake genera such as *Cenaspis* (Campbell et al. 2018), *Metlapilcoatlus* (Campbell et al. 2019) and *Desertum* (Blair et al. 2022).

Because of the efforts invested to study the Mexican herpetofauna by numerous scientists with varying levels of experience, Mexico is known as one of the richest countries with respect to this group of vertebrates (Wilson et al. 2010, 2013a, b; Johnson et al. 2017). Considering the above information, the main goal of this study is to provide an updated list of the species that comprise the Mexican herpetofauna (as of April 2023). The update of amphibian and reptile species for Mexico (and by states) is necessary due to the dynamics that have been taking place in the descriptions of new species for science, as well as recent taxonomic changes and new reports of exotic species, which collectively increase the number of species in these groups. The latest studies where the lists of species presented by Balderas-Valdivia and González-Hernández (2021) and Balderas-Valdivia et al. (2022) with only native species, however, in the present study the most updated list is shown both in number of species (native and introduced) and in taxonomic update.

## ﻿Materials and methods

The updated species lists are based on the following:

1. Publications such as Herpetology of Mexico (Smith and Taylor 1966), and studies at the regional (Pérez-Higareda and Smith 1991; Ramírez-Bautista 1994; Uribe-Peña et al. 1999; among others), state (Álvarez del Toro 1982; Vázquez-Díaz and Quintero-Díaz 1997, 2005; Lemos-Espinal and Smith 2009, 2016; Ramírez-Bautista et al. 2009, 2010, 2014; Dixon and Lemos-Espinal 2010; Alvarado-Díaz et al. 2013; Lemos-Espinal and Dixon 2013; Carbajal-Márquez and Quintero-Díaz 2016; Lemos-Espinal et al. 2016, 2017, 2018a, b, 2019; Nevárez-de los Reyes et al. 2016; Terán-Juárez et al. 2016; Woolrich-Piña et al. 2016, 2017; Cruz-Sáenz et al. 2017; González-Sánchez et al. 2017; Palacios-Aguilar and Flores-Villela 2018; Ramírez-Bautista et al. 2020; Rhodin et al. 2021; Barragán-Vázquez et al. 2022; Cruz-Elizalde et al. 2022; Leyte-Manrique et al. 2022; among others), and country levels (Flores-Villela 1993a; Flores-Villela and Canseco-Márquez 2004; Liner 2007; Wilson and Johnson 2010; Wilson et al. 2013a, b; Flores-Villela and García-Vázquez 2014; Parra-Olea et al. 2014; Mata-Silva et al. 2015; Johnson et al. 2015, 2017; Balderas-Valdivia and González-Hernández 2021; Mata-Silva et al. 2021; Balderas-Valdivia et al. 2022; Smith and Lemos-Espinal 2022);
2. historical records (databases) compiled by the authors, which were searched from 1940 to date;
3. records obtained in specialized journals, such as Mesoamerican Herpetology, ZooKeys, Zootaxa, Revista Mexicana de Biodiversidad, Herpetological Review, and Amphibian & Reptile Conservation, as well as publications of newly described species for the country (until 30 April 2023);
4. databases generated from projects undertaken by the Comisión Nacional para el Conocimiento y Uso de la Biodiversidad (CONABIO 2019; https://www.gob.mx/conabio); and
5. records of amphibians and reptiles from Mexico obtained from the Global Biodiversity Information Facility (GBIF 2019; https://www.gbif.org/), and authenticated by geographic distribution records from previously published literature.

All taxonomic changes and the identification of the species were based on the most recent taxonomic revisions (Martínez-Méndez and Méndez-De la Cruz 2007; Poe 2013; Wilson et al. 2013a, b; Ruane et al. 2014; Meza-Lázaro and Nieto-Montes de Oca 2015; Montanucci 2015; Rovito et al. 2015; Duellman et al. 2016; Leavitt et al. 2017; Canseco-Márquez et al. 2018; Campbell et al. 2019; Campillo-García et al. 2021; Gutiérrez-Rodríguez et al. 2021; Everson et al. 2021; Hernández-Jiménez et al. 2021; Köhler 2021; Blair et al. 2022; Hernández-Austria et al. 2022; Pérez-Ramos and Luja-Molina 2022; Reyes-Velasco et al. 2022; Devitt et al. 2023; Pérez-Ramos 2023). The scientific names of amphibians were updated according to Amphibian Species of the World (Frost 2023, https://www.amnh.org), and for reptiles, based on the Reptile Database website (Uetz et al. 2023; http://www.reptile-database.org/).

## ﻿Results

### ﻿Composition of the current Mexican herpetofauna

Our results show that the current native herpetofauna of Mexico is comprised as follows: amphibians = 16 families, 58 genera, and 430 species (Suppl. material 1); and reptiles = 44 families, 155 genera, and 975 species (Suppl. material 2). For both groups the totals = 60 families, 213 genera, and 1,405 species (see Suppl. materials 1, 2; Table 1).

**Table 1.** Composition of the native herpetofauna of Mexico, including number of endemic species and percentage of endemism.

Orders	Families	Genera	Species	Endemic Species	Percentage of Endemism (%)
Anura	11	37	270	168	62.2
Caudata	4	19	157	131	83.4
Gymnophiona	1	2	3	1	33.3
**Subtotals**	**16**	**58**	**430**	**300**	**69.8**
Crocodylia	2	2	3	–	–
Squamata	31	135	921	566	61.3
Testudines	10	18	51	20	39.2
**Subtotals**	**43**	**155**	**975**	**586**	**60.1**
**Totals**	**59**	**213**	**1405**	**886**	**63.1**

### ﻿Increase in the total size of the native Mexican herpetofauna from 1577 to the present

**Figure 1.**
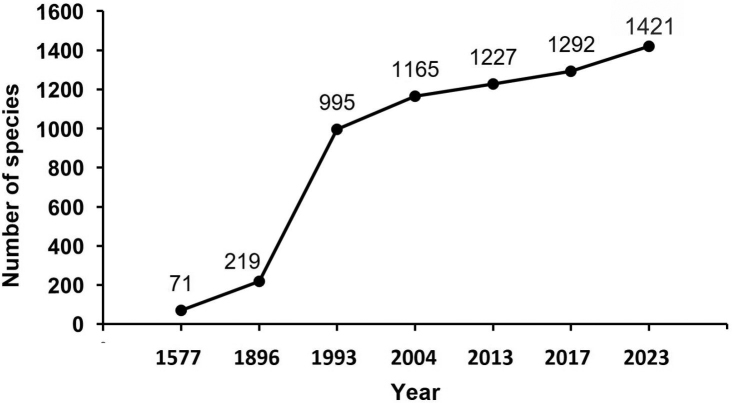
Cumulative curve of the herpetofauna recorded in Mexico. The years correspond to studies carried out by Hernández (1577, published in 1959), Dugès (1896), Flores-Villela (1993a), Flores-Villela and Canseco-Márquez (2004), Wilson et al. (2013a, b), Johnson et al. (2017), and this study.

The size of the Mexican herpetofauna has increased ca. 20.1-fold in the period from 1577 to the present (from 71 species to 1,421; see Fig. 1). During the ensuing 319 years, the number of species has increased ca. 3.1-fold, from 71 to 219 species or ca. 310%. The increase over this three-century period was 148 species. The largest increase during the history of the study of the Mexican herpetofauna transpired from 1896 to 1993 (97 years), when the number of species rose from 219 to 995 (or 776 species), a 3.5-fold increase or ca. 350%. Since 1993, however, the rate of increase has slowed somewhat. From 1993 (995 species) to 2004 (1,165 species), the increase was 170 species; from 2004 (1,165 species) to 2013 (1,227 species) 62 species were added; and from 2013 (1,227 species) to 2017 (1,292 species) there were 65 additional species. Finally, from 2017 to the present (2023), the numbers increased from 1,292 species to 1,421, in this study 129 species are added (Fig. 1).

### ﻿Increase in the size of the non-native herpetofauna in Mexico

Currently, 16 species of non-native or exotic amphibians and reptiles have been reported from Mexico (González-Sánchez et al. 2021; Tepos-Ramírez et al. 2022), including two amphibians (*Eleutherodactylusplanirostris* and *Xenopuslaevis*) and 14 reptiles (*Anolisallisoni*, *A.carolinensis*, *A.cristatellus*, *A.sagrei*, *Gehyramutilata*, *Hemidactylusfrenatus*, *H.garnotii*, *H.mabouia*, *H.turcicus*, *Lepidodactyluslugubris*, *Tarentolamauritanica*, *Sphaerodactylusargus*, *Virgotyphlopsbraminus*, and *Graptemyspseudogeographica*).

### ﻿Endemism of the current Mexican herpetofauna

The percentage of species endemism of the Mexican herpetofauna is very high at 63.1% (Table 1). By groups, amphibians represent the highest percentage of endemism, with 69.8% (with 300 species), and a somewhat lower number in reptiles at 60.1% (586 species).

### ﻿Species richness among states

**Figure 2.**
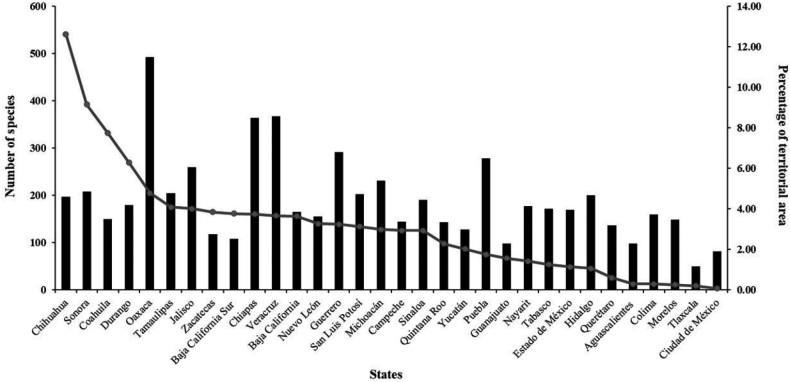
Number of herpetofaunal species in Mexico by state (bars), and proportion of territorial surface (line).

**Figure 3.**
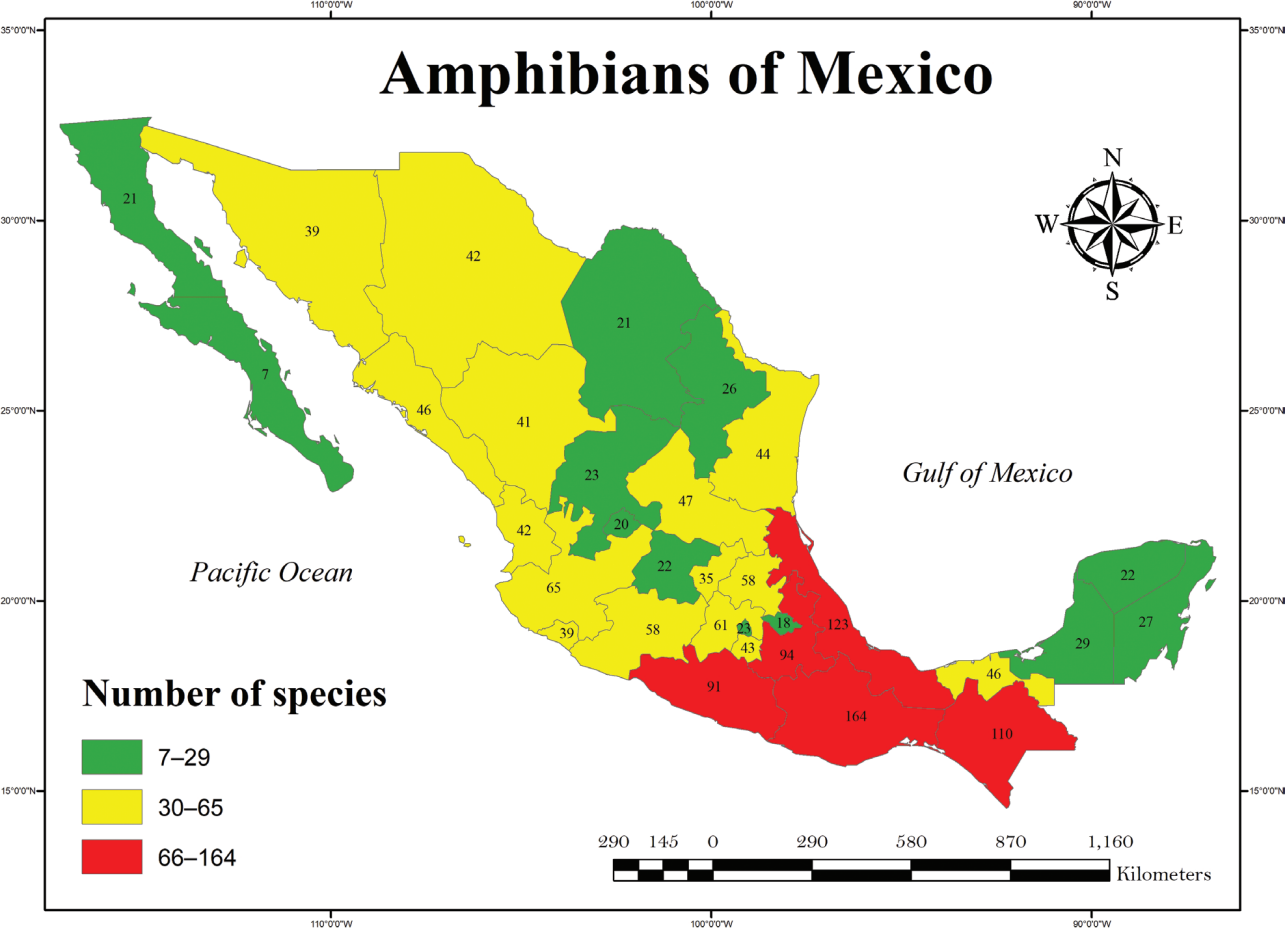
Number of species of amphibians in Mexico by state. The color scale represents the species numbers from low (green) to high (red).

**Figure 4.**
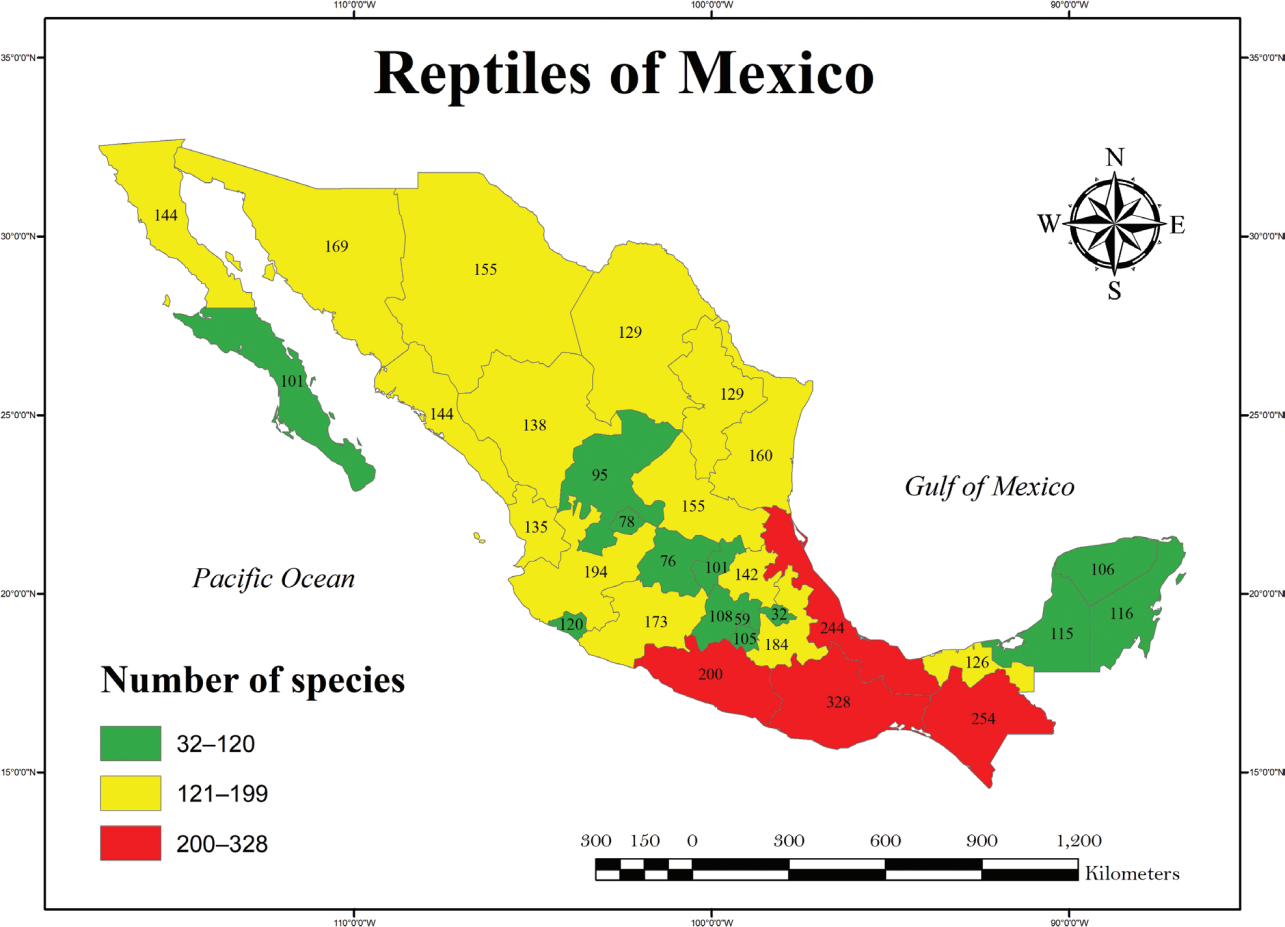
Number of species of reptiles in Mexico by state. The color scale represents the species numbers from low (green) to high (red).

Currently, of the 32 federal entities, three states in southeastern Mexico (Oaxaca, Veracruz, and Chiapas) maintain the highest richness levels (Fig. 2), i.e., 492 (34.6% of Mexican herpetofauna), 367 (25.8%), and 364 (25.6%), respectively (Figs 3, 4, Suppl. material 3). These states are followed by Guerrero (291, 20.5%), Puebla (278, 19.6%), Jalisco (259, 18.2%), Michoacán (231, 16.3%), Sonora (208, 14.6%), Tamaulipas (204, 14.4%), San Luis Potosí (202, 14.2%) and Hidalgo (200, 14.1%), whereas the states with the lowest number of species are Zacatecas (118, 8.3%), Baja California Sur (108, 7.6%), Guanajuato (98, 6.9%), Aguascalientes (98, 6.9%), Ciudad de México (82, 5.8%), and Tlaxcala (50, 3.5%; Figs 2–4, Suppl. material 3).

### ﻿Areal extension by state and species richness

The size of the 32 federal entities in Mexico varies considerably from that of Ciudad de México (1,494.6 km^2^) to that of Chihuahua (247,412.5 km^2^), of which the latter is 165.5 times larger than the former (Suppl. material 3). Obviously, the percentage of the territorial area occupied by these 32 entities also varies considerably (from 0.08 to 12.62%). These percentage values fall into a number of categories, including 0.08–1.00% (Aguascalientes, Colima, Ciudad de México, Morelos, Querétaro, Tlaxcala), 1.01–3.00 (Campeche, Guanajuato, Hidalgo, Estado de México, Michoacán, Nayarit, Puebla, Quintana Roo, Sinaloa, Tabasco, and Yucatán), 3.01–6.00 (Baja California, Baja California Sur, Chiapas, Guerrero, Jalisco, Nuevo León, Oaxaca, San Luis Potosí, Tamaulipas, Veracruz, and Zacatecas), and 6.01 and above (Chihuahua, Coahuila, Durango, and Sonora). By territorial area, the largest states are Chihuahua, Sonora, Coahuila, and Durango, all located in northern and north-central Mexico (Fig. 2). These states, however, exhibit relatively low species richness (197, 208, 150, and 179, respectively) compared to several smaller states like Oaxaca, Chiapas, Guerrero, Veracruz, Puebla, and Jalisco (see values in section above; Fig. 2; Suppl. material 3).

The lowest values for amphibian species (7–29) are found in the two states of the Peninsula of Baja California, a north-central swath of states that include Coahuila, Nuevo León, Zacatecas, Aguascalientes, and Guanajuato, two small states in central Mexico, Ciudad de México, and Tlaxcala, and the Yucatan Peninsula states, Campeche, Yucatán, and Quintana Roo (Fig. 3). The next highest category (30–65) includes the northwestern and western states of Sonora, Chihuahua, Durango, Sinaloa, and Nayarit, Jalisco, Colima, and Michoacán, and the northeastern states, of Tamaulipas and San Luis Potosí, the east-central states of Hidalgo and Querétaro, the central Mexican states including state of México and Morelos, and southeast state of Tabasco. The highest category (66–164) is comprised of a group of states in southern Mexico, including Guerrero, Puebla, Veracruz, Oaxaca, and Chiapas.

According to the number of species of reptiles by state the resulting categories are somewhat different than those for the amphibians, since the lowest rank (32–120) is occupied by the states of Baja California Sur, a smaller central Mexican swath that includes Zacatecas, Aguascalientes, Guanajuato, Querétaro, Estado de México, Ciudad de México, Tlaxcala, and Morelos, the state of Colima, and the states of the Yucatan Peninsula (Campeche, Yucatán, and Quintana Roo) (Fig. 4). The medium category (121–199) comprises the remaining states in northern and central Mexico, and southeastern state of Tabasco. The high category (200–328) consists of a single cluster of states that includes Guerrero, Veracruz, Oaxaca, and Chiapas. The general pattern for the distribution of reptile species in Mexico is a low level of species richness in Baja California Sur, the central Mexican swath of states and Colima, and the states of Campeche, Yucatán, and Quintana Roo, a medium level in the remaining states in northern and central Mexico, and the southeastern state of Tabasco, and lastly, a high level in the southern and eastern states of Guerrero, Veracruz, Oaxaca, and Chiapas.

## ﻿Discussion

The richness of amphibian and reptile species in Mexico has been the subject of study for several decades, generating various works listing the herpetofauna at the country level. In this study, we update the list of species to 1,421, of which 1,405 are native species, and 16 are non-native. The number of native species we record in this study is higher compared to that of recent works by Balderas-Valdivia and González-Hernández (2021; 1,389 species), and Balderas-Valdivia et al. (2022; 1,393 species) for the herpetofauna of Mexico, based on new discoveries.

The high species richness and endemism of both groups stands out compared to that of other groups of vertebrates in Mexico (Smith and Lemos-Espinal 2022), since Johnson et al. (2017) noted that the country of Mexico contains the fifth largest amphibian fauna and the second largest reptile fauna in the world. At that time, the size of the Mexican amphibian fauna was smaller than only those of Brazil, Colombia, Peru, and Ecuador, and the Mexican reptile fauna was smaller than only that of Australia.

The brief historical review shown in the introduction of this work highlights the continuous study of the herpetofauna to date, since it began with the acknowledgement of the amphibian and reptile fauna by the Aztecs, which later increased with the Spanish conquest of the Americas (Martín del Campo 1979). Although Europeans made this conquest in the 17^th^ century, the principal impulse that led to our current knowledge of the Mexican herpetofauna did not occur until the second half of the 19^th^ century and was made possible through the contributions of Smith and Smith (1976) and Flores-Villela (1993b). By late 19^th^ century the number of species in the country was ca. 219, but by the end of 20^th^ century it had increased to 995 species (Fig. 1). This dynamic increase was driven primarily by the continuous work of Mexican (Álvarez-del Toro 1982; Flores-Villela and Canseco-Márquez 2004; Lemos-Espinal and Smith 2009, 2016; Lemos-Espinal and Dixon 2013; Ramírez-Bautista et al. 2014; Mata-Silva et al. 2015; Lemos-Espinal et al. 2016) and American herpetologists (Wilson et al. 2010; 2013a, b; Johnson et al. 2017). At the beginning of the 21^st^ century, the increased herpetofaunal species richness resulted from new state records (Badillo-Saldaña et al. 2019; Tepos-Ramírez et al. 2022) and the descriptions of new species (Flores-Villela and Canseco-Márquez 2004; Lara-Tufiño et al. 2016; Campbell et al. 2018; García-Castillo et al. 2018; García-Vázquez et al. 2018a, b; Barragán-Reséndiz et al. 2022; Flores-Villela et al. 2022a, b; Nieto-Montes de Oca et al. 2022; Grünwald et al. 2023). All these studies increased the herpetofauna to a current total of 1,421 species.

Based on this number, the herpetofauna of Mexico has been analyzed by authors in other related fields, including conservation (Wilson et al. 2010) and biogeography (Wilson et al. 2013a, b; Johnson et al. 2017). The species endemism value of 63.1% is similar to that reported by Johnson et al. (2017; 61.2%) but is higher than that reported for Central America by Mata-Silva et al. (2019; 56.9%). By comparing the 2017 value and the present values for Mexican endemism, evidently the species added to the herpetofauna for ca. the last five years have been reported primarily as endemic to the country, as opposed to reported for Mexico from outside the country.

The states in southeastern Mexico, including Oaxaca, Chiapas, and Veracruz, have shown the highest amount of species richness (Figs 2–4; Suppl. material 3; Balderas-Valdivia and González-Hernández 2021; Balderas-Valdivia et al. 2022), and these values are partly supported by such factors as environmental heterogeneity, climate, vegetation types, and their influence on biogeographic regions, as documented by other studies (Ochoa-Ochoa et al. 2014; Ramírez-Bautista et al. 2014; Cruz-Elizalde et al. 2016, among others). Note that states in the northern part of the country, which are located within a longer territorial extension, have a low number of species (Fig. 2; Suppl. material 3). These states are also characterized by an environmental homogeneity in vegetation types and other physiographic characteristics (Lemos-Espinal and Dixon 2013; Lemos-Espinal et al. 2016). For states with a small territorial extension, such as Guerrero, Puebla, Jalisco, Tamaulipas, San Luis Potosí, Michoacán, Nayarit, and Hidalgo, their high species richness might be explained not only by their environmental heterogeneity, but also by the convergence of biogeographic zones (Ochoa-Ochoa et al. 2014; Ramírez-Bautista et al. 2014; Palacios-Aguilar and Flores-Villela 2018; Smith and Lemos-Espinal 2022). These results have been explained for other studies on the Mexican herpetofauna (Smith and Lemos-Espinal 2022), as well as in other biological groups such as mammals (Ceballos et al. 1998) or birds (Ramírez-Albores et al. 2020). In these studies, the authors show a trend in which the tropical zones, and mainly in the Mesoamerican region and low latitudes, the richness of species increases. This trend is also supported by the complex orography and diverse environments in these areas (Ochoa-Ochoa et al. 2014).

Future studies on systematics (Campillo-García et al. 2021), regional checklists, and isolated geographic records will keep increasing the number of species. In this sense, the continuous study of the herpetofauna conjugates on plans regarding conservation, since a high percentage of herpetofaunal species are endemic to the country, and many are regarded as threatened on account of human activities, e.g., overpopulation, habitat fragmentation, pollution, agriculture, among other factors (Wilson et al. 2010; Johnson et al. 2017).

## ﻿Additional information

### Author ORCIDs

Aurelio Ramírez-Bautista https://orcid.org/0000-0003-2948-0178

Raciel Cruz-Elizalde https://orcid.org/0000-0001-9630-7063

Christian Berriozabal-Islas https://orcid.org/0000-0002-3739-3990

Uriel Hernández-Salinas https://orcid.org/0000-0001-9630-7063

Larry David Wilson https://orcid.org/0000-0003-4969-0789

Jerry D. Johnson https://orcid.org/0000-0002-2135-4866

Carlos Jesús Balderas-Valdivia https://orcid.org/0000-0002-1378-7610

Adriana J. X. González-Hernández https://orcid.org/0000-0002-6445-3338

Vicente Mata-Silva https://orcid.org/0000-0001-8123-1844

### Data availability

All of the data that support the findings of this study are available in the main text or Supplementary Information.

## Supplementary materials

Supplementary material 1 List of 430 taxa of native (and 2 non-native) amphibians of Mexico Data type Species list (xlsx. file) https://binary.pensoft.net/file/862856This dataset is made available under the Open Database License (http://opendatacommons.org/licenses/odbl/1.0/). The Open Database License (ODbL) is a license agreement intended to allow users to freely share, modify, and use this Dataset while maintaining this same freedom for others, provided that the original source and author(s) are credited.Aurelio Ramírez-Bautista, Lizzeth A. Torres-Hernández, Raciel Cruz-Elizalde Christian Berriozabal-Islas, Uriel Hernández-Salinas, Larry David Wilson, Jerry D. Johnson, Louis W. Porras, Carlos Jesús Balderas-Valdivia, Adriana J. X. González-Hernández, Vicente Mata-Silva

Supplementary material 2 List of 975 taxa of native (and 14 non-native) reptiles of Mexico Data type Species list (xlsx. file) https://binary.pensoft.net/file/862857This dataset is made available under the Open Database License (http://opendatacommons.org/licenses/odbl/1.0/). The Open Database License (ODbL) is a license agreement intended to allow users to freely share, modify, and use this Dataset while maintaining this same freedom for others, provided that the original source and author(s) are credited.Aurelio Ramírez-Bautista, Lizzeth A. Torres-Hernández, Raciel Cruz-Elizalde Christian Berriozabal-Islas, Uriel Hernández-Salinas, Larry David Wilson, Jerry D. Johnson, Louis W. Porras, Carlos Jesús Balderas-Valdivia, Adriana J. X. González-Hernández, Vicente Mata-Silva

Supplementary material 3 Territorial area, percentage territorial of county, and total number of species of amphibians, reptiles, and herpetofauna of each state in Mexico Data type table (docx. file) https://binary.pensoft.net/file/862858This dataset is made available under the Open Database License (http://opendatacommons.org/licenses/odbl/1.0/). The Open Database License (ODbL) is a license agreement intended to allow users to freely share, modify, and use this Dataset while maintaining this same freedom for others, provided that the original source and author(s) are credited.Aurelio Ramírez-Bautista, Lizzeth A. Torres-Hernández, Raciel Cruz-Elizalde Christian Berriozabal-Islas, Uriel Hernández-Salinas, Larry David Wilson, Jerry D. Johnson, Louis W. Porras, Carlos Jesús Balderas-Valdivia, Adriana J. X. González-Hernández, Vicente Mata-Silva
