# Supplementary material for: ﻿An updated list of the Mexican herpetofauna: with a summary of historical and contemporary studies
Source: Zookeys. 2023 Jun 12;1166:287–306. doi: 10.3897/zookeys.1166.86986 (PMC10280392; doi:10.3897/zookeys.1166.86986)
Supplement: Supplementary material 3 — Territorial area, percentage territorial of county, and total number of species of amphibians, reptiles, and herpetofauna of each state in Mexico [file zookeys-1166-287_article-86986__-s003.docx]

**Supplementary file 3.** Territorial area, percentage of territorial area, and total number of species of amphibians, reptiles, and herpetofauna of each state in Mexico.

| **States** | **Territorial area (km^2^)** | **Percentage of territorial area** | **Number of species of amphibians** | **Number of species of reptiles** | **Number of species of herpetofauna** |
| --- | --- | --- | --- | --- | --- |
| Aguascalientes | 5,615.70 | 0.29 | 20 | 78 | 98 |
| Baja California | 71,450.40 | 3.64 | 21 | 144 | 165 |
| Baja California Sur | 73,909.40 | 3.77 | 7 | 101 | 108 |
| Campeche | 57,507.50 | 2.93 | 29 | 115 | 144 |
| Coahuila | 151,594.50 | 7.73 | 21 | 129 | 150 |
| Colima | 5,626.90 | 0.29 | 39 | 120 | 159 |
| Chiapas | 73,311.00 | 3.74 | 110 | 254 | 364 |
| Chihuahua | 247,412.50 | 12.62 | 42 | 155 | 197 |
| Ciudad de México | 1,494.60 | 0.08 | 23 | 59 | 82 |
| Durango | 123,364.20 | 6.29 | 41 | 138 | 179 |
| Guanajuato | 30,606.70 | 1.56 | 22 | 76 | 98 |
| Guerrero | 63,595.90 | 3.24 | 91 | 200 | 291 |
| Hidalgo | 20,821.40 | 1.06 | 58 | 142 | 200 |
| Jalisco | 78,595.90 | 4.01 | 65 | 194 | 259 |
| Estado de México | 22,351.50 | 1.14 | 61 | 108 | 169 |
| Michoacán | 58,598.70 | 2.99 | 58 | 173 | 231 |
| Morelos | 4,878.90 | 0.25 | 43 | 105 | 148 |
| Nayarit | 27,856.50 | 1.42 | 42 | 135 | 177 |
| Nuevo León | 64,156.30 | 3.27 | 26 | 129 | 155 |
| Oaxaca | 93,757.40 | 4.78 | 164 | 328 | 492 |
| Puebla | 34,309.10 | 1.75 | 94 | 184 | 278 |
| Querétaro | 11,690.60 | 0.60 | 35 | 101 | 136 |
| Quintana Roo | 44,705.50 | 2.28 | 27 | 116 | 143 |
| San Luis Potosí | 61,138.00 | 3.12 | 47 | 155 | 202 |
| Sinaloa | 57,365.40 | 2.93 | 46 | 144 | 190 |
| Sonora | 179,355.10 | 9.15 | 39 | 169 | 208 |
| Tabasco | 24,731.40 | 1.26 | 46 | 126 | 172 |
| Tamaulipas | 80,249.30 | 4.09 | 44 | 160 | 204 |
| Tlaxcala | 3,997.20 | 0.20 | 18 | 32 | 50 |
| Veracruz | 71,823.50 | 3.66 | 123 | 244 | 367 |
| Yucatán | 39,524.10 | 2.02 | 22 | 106 | 128 |
| Zacatecas | 75,275.40 | 3.84 | 23 | 95 | 118 |
